# Cost–Effectiveness Analysis of Dapagliflozin Plus Standard Treatment for Patients With Type 2 Diabetes and High Risk of Cardiovascular Disease in China

**DOI:** 10.3389/fpubh.2022.936703

**Published:** 2022-07-13

**Authors:** Kaiyu Huang, Yao Wang, Sijia Sun, Qian Zhu, Weifeng Zhou, Jiatao Liu, Dongchun Zhu, Xuefeng Xie

**Affiliations:** ^1^Key Laboratory of Anti-inflammatory and Immunopharmacology, Department of Clinical Pharmacy, Ministry of Education, Anhui Medical University, Hefei, China; ^2^The Key Laboratory of Major Autoimmune Diseases, Anhui Institute of Innovative Drugs, School of Pharmacy, Anhui Medical University, Hefei, China; ^3^Department of Clinical Medicine, Anhui Medical College, Hefei, China; ^4^Department of Pharmacy, First Affiliated Hospital of Anhui Medical University, Hefei, China

**Keywords:** diabetes, cardiovascular disease, dapagliflozin, Markov model, cost-effectiveness

## Abstract

**Purpose:**

To evaluate the long-term cost-effectiveness of dapagliflozin, in addition to standard treatment, for the treatment of adult patients with type 2 diabetes (T2DM) at high cardiovascular risk from the Chinese healthcare system perspective.

**Methods:**

A decision-analytic Markov model with one-year cycles was developed to evaluate the health and economic outcomes in patients with T2DM and high risk of cardiovascular disease (CVD) treated with standard treatment and dapagliflozin plus standard treatment for 30 years. Clinical data, cost, and utility data were extracted from databases or published literature. Quality-adjusted life-years (QALYs), costs (€/¥ 2021) as well as incremental cost-effectiveness ratios (ICERs) were calculated. Deterministic and probabilistic sensitivity analyses were performed to assess the uncertainty in the results.

**Results:**

Compared with standard treatment, dapagliflozin plus standard treatment was predicted to result in an additional 0.25 QALYs (12.26 QALYs vs. 12.01 QALYs) at an incremental cost of €4,435.81 (¥33,875.83) per patient. The ICER for dapagliflozin plus standard treatment vs. standard treatment was €17,742.07 (¥135,494.41) per QALY gained, which was considered cost-effective in China compared to three times the GDP per capita in 2021 (€31,809.77/¥242,928). The deterministic and probabilistic sensitivity analyses showed the base-case results to be robust.

**Conclusions:**

The study suggests that, from the perspective of the Chinese health system, dapagliflozin plus standard treatment is a cost-effective option for patients with T2DM at high cardiovascular risk. These findings may help clinicians make the best treatment decisions for patients with T2DM at high cardiovascular risk.

## Introduction

Type 2 diabetes mellitus has become a serious global public health problem. According to the International Diabetes Federation (IDF) Diabetes Atlas 10th edition, about 536.6 million people (20 to 79 years old) had diabetes worldwide in 2021, and the prevalence rate of diabetes was 10.5%. China has the largest number of diabetic patients in the world (140.9 million), with an estimated T2DM prevalence of 10.6% in the adult population (1). More and more evidence shown that diabetic patients have higher risk of cardiovascular disease (2, 3). And the risk of cardiovascular disease increased by 2–4 times compared with non-diabetic patients (4). Otherwise, cardiovascular disease is the leading cause of death in patients with type 2 diabetes (5). An epidemiological study shown that cardiovascular disease accounted for about 43.2% of China's diabetes related deaths. Ischemic heart disease (IHD) accounted for about 18.7%, stroke accounted for about 17.1%, and about 7.3% of patients died of other cardiovascular diseases (6).

Sodium-glucose cotransporter 2 (SGLT-2) inhibitors is an oral hypoglycemic drug that have received great attention in recent years. SGLT-2 inhibitors can reduce the renal glucose threshold of T2DM patients and promote the excretion of excess glucose in urine by highly selectively inhibiting the reabsorption of glucose by SGLT-2 protein in the early portion of the proximal renal tubule (the S1 segment), so as to reduce the blood glucose level (7). Relevant studies have shown that SGLT-2 inhibitors can not only reduce blood glucose, but also improve endothelial dysfunction, improve ventricular loading conditions, improve cardiac metabolism and bioenergetics, inhibit cardiac fibrosis, inhibit adipocytokines, reduce blood pressure and have direct effects on Na^+^/H^+^ exchange in the myocardium, so as to have a protective effect against cardiovascular disease (8, 9). Therefore, in the guidelines and consensus on diabetes prevention and treatment of various countries, SGLT-2 inhibitors are recommended for the treatment of T2DM patients with established cardiovascular disease or high risk of cardiovascular disease. Dapagliflozin is the first SGLT-2 inhibitor listed in China, DECLARE–TIMI 58 trial showed that on the basis of standard treatment, the combination of dapagliflozin can significantly reduce the incidence of cardiovascular death or hospitalization for heart failure in T2DM patients with confirmed atherosclerotic cardiovascular disease (ASCVD) or multiple risk factors of ASCVD (10). In the DECLARE–TIMI 58 trial, patients who were assigned to dapagliflozin group received standard treatment and an oral dose of 10mg dapagliflozin daily, patients in placebo group received standard treatment and orally taken placebo 10mg daily. The standard treatment (other than an open-label SGLT2 inhibitor, pioglitazone, or rosiglitazone) was at the discretion of the treating physician. Patients were followed up every 6 months until the trial was completed. The use of dapagliflozin improved blood glucose levels and cardiovascular clinical outcomes in T2DM patients with high cardiovascular risk.

Diabetes has brought heavy financial burden to the world. The International Diabetes Federation estimates that the global health expenditure caused by diabetes in 2021 was about 966 billion dollars (1). The cardiovascular complications of diabetes greatly increase the cost of treatment for diabetes. Systematic review of the economic burden of cardiovascular disease in patients with type 2 diabetes shown that the cost of cardiovascular disease was about 20–49% of the direct cost of treatment for type 2 diabetes (11). Previous researches have evaluated the pharmacoeconomic profiles of dapagliflozin for patients with T2DM and high cardiovascular risk in the USA, Thailand, Greece and UK, using Markov model, Cardiff model or discrete event simulation model (12–15). However, due to the significant differences between national health systems and medical costs, these results can not apply to Chinese. Currently, there was lack of study based on China's real world data to evaluate the cost-effectiveness of dapagliflozin in the treatment of T2DM patients with high risk of cardiovascular disease. The objective of this study was to evaluate the long-term cost-effectiveness of dapagliflozin, as an add-on to standard treatment, for the treatment of patients with T2DM at high cardiovascular risk from the Chinese healthcare system perspective, and provided useful information for the rational drug use of patients with T2DM and high cardiovascular risk.

## Materials and Methods

### Patient Populations

This study assumed that the baseline data of modeled population was sourced from the DECLARE-TIMI 58 clinical trial. DECLARE-TIMI 58 clinical trial was a randomized, controlled, double-blind, multicenter phase three clinical trial, which evaluated 17,160 patients, including those from China. The median follow-up of the trial was 4.2 years. In brief, all patients had type 2 diabetes, glycosylated hemoglobin (HbA1c) was in a range between 6.5 and 12%, and a creatinine clearance of 60 ml/min or more, and the patient was at least 40 years old. Furthermore, all patients were at high risk of cardiovascular disease, which was defined as having multiple risk factors of atherosclerotic cardiovascular disease or had diagnosed as atherosclerotic cardiovascular disease (10). The patients did not have cardiovascular events such as myocardial infarction, angina pectoris, stroke and heart failure 8 weeks before enrollment.

### Intervention and Comparator

The patients in the intervention group received 10 mg of dapagliflozin daily as an add-on to standard treatment, and patients in the control group received only standard treatment (16). Standard treatment was defined as best available hypoglycemic therapy determined by doctors, and on the basis of the DECLARE-TIMI 58 baseline characteristics of the patients, the hypoglycemic drugs of standard treatment include insulin, metformin, sulfonylureas, DPP-4 inhibitors and GLP-1 receptor agonists. The proportions of patients receiving these hypoglycemic drugs were similar between the two groups.

### Model Structure

**Figure 1:**
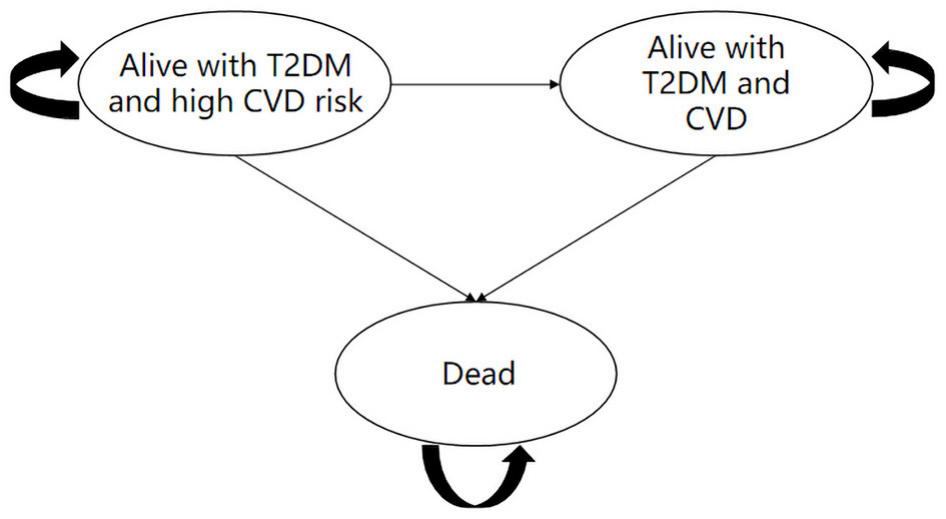
Diagram of the Markov state transition model and health states of dapagliflozin plus standard vs. standard treatment. T2DM, type 2 diabetes mellitus; CVD, cardiovascular disease.

**Figure 2:**
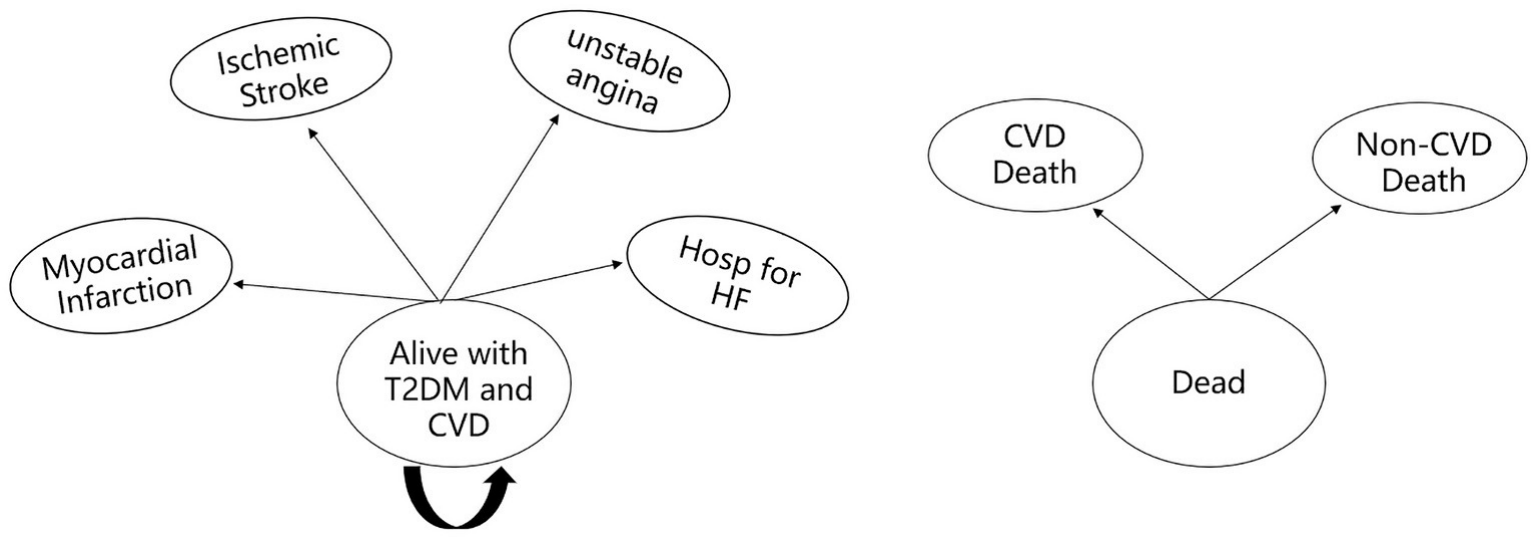
Diagram of the “Alive with T2DM and CVD” and “Dead” health states. Hosp for HF, hospitalization for heart failure; CVD death, death from cardiovascular cause; Non-CVD death, death from non-cardiovascular cause.

A Markov model was established using TreeAge 2019 software to evaluate the cost and health outcomes of standard treatment and dapagliflozin plus standard treatment in patients with type 2 diabetes and high risk of cardiovascular disease. The model comprised three health states: “Alive with T2DM and high cardiovascular risk,” “Alive with T2DM and CVD” and “Dead” (Figure 1). When patients in the “Alive with T2DM and high cardiovascular risk” health state, they did not have myocardial infarction, ischemic stroke, unstable angina and hospitalization for heart failure. Patients in the health status of “Alive with T2DM and CVD” were at risk for suffering a combination of CVD events (including myocardial infarction, ischemic stroke, unstable angina and hospitalization for heart failure), and death from cardiovascular or non-cardiovascular causes (17) (Figure 2).

The study assumed that the initial health status of all patients is “Alive with T2DM and high cardiovascular risk.” In the process of time, the patients might remain in their assigned health state or transferred to a new health state. It should be noted that patients cannot return to their previous health state. According to the characteristics of the disease and the existing literatures research results, this study set the Markov cycle length as 1 year (17), and simulated the cost and health outcomes of patients treated with standard treatment or dapagliflozin plus standard treatment for 30 years (17, 18). Base on the data extracted from the DECLARE-TIMI 58 clinical trial, the main model outcomes were average quality-adjusted life-years (QALYs) and costs of 17,160 patients in the two groups, and the incremental cost effectiveness ratio (ICER) was used to judge whether the therapeutic schedule is economical. According to the China Guidelines for Pharmacoeconomic Evaluations (2020 edition), the cost and utility values were discounted at a rate of 5% per year, and three times China's per capita gross domestic product (GDP) in 2021 (€31,809.77/¥242,928) was used as the willingness to pay (WTP) threshold (19).

### Input Parameters

#### Transition Probabilities

We obtained the transition probabilities of CVD events rate, mortality of cardiovascular cause and mortality of non-cardiovascular cause from DECLARE-TIMI 58 clinical trial, which evaluated the cardiovascular morbidity and mortality between both groups in patients with T2DM at high cardiovascular risk over 4.2 years of follow-up (10). This study used the cardiovascular mortality and non-cardiovascular mortality of ASCVD subgroup analysis of DECLARE-TIMI 58 clinical trial to replace mortality of T2DM patients with ischemic stroke or unstable angina. Mortality in patients with MI and heart failure was obtained from subgroup analysis of patients with previous MI and heart failure in the DECLARE-TIMI 58 clinical trial (20, 21). Since the subgroup analysis did not give the mortality of non-cardiovascular causes in patients with T2DM and heart failure/MI, we used the all-cause mortality minus the cardiovascular causes mortality to obtain the non-cardiovascular causes mortality. We used the formula by Briggs et al. (22) to calculate transition probabilities from the event rates: *r* = −(1/4.2) ^*^ln(1–R), where *r* is the 1-year rate and R is the 4.2-year rate, and then use the formula *t*_p_ = 1–e^−*r*^ to calculate the 1-year transition probability. All transition probabilities were shown in Table 1.

**Table 1:** Transition probabilities.

**Parameter**	**Point estimate**	**Variation range**		**References**

		**Lower**	**Upper**	
Dapagliflozin plus standard treatment				
T2DM with CVD high risk to MI	0.0111	0.0101	0.0122	(10)
T2DM with CVD high risk to ischemic stroke	0.0066	0.0058	0.0075	(10)
T2DM with CVD high risk to unstable angina	0.0076	0.0068	0.0086	(10)
T2DM with CVD high risk to HHF	0.0059	0.0052	0.0068	(10)
T2DM with CVD high risk to CVD death	0.0069	0.0061	0.0078	(10)
T2DM with CVD high risk to Non-CVD death	0.0059	0.0052	0.0068	(10)
T2DM with MI to CVD death	0.0119	0.0096	0.0146	(20)
T2DM with MI to Non-CVD death	0.0090	0.0071	0.0114	(20)
T2DM with ischemic stroke to CVD death	0.0107	0.0091	0.0125	(10)
T2DM with ischemic stroke to Non-CVD death	0.0069	0.0057	0.0084	(10)
T2DM with unstable angina to CVD death	0.0107	0.0091	0.0125	(10)
T2DM with unstable angina to Non-CVD death	0.0069	0.0057	0.0084	(10)
T2DM with HHF to CVD death	0.0198	0.0159	0.0246	(21)
T2DM with HHF to Non-CVD death	0.0106	0.0079	0.0143	(21)
Standard treatment				
T2DM with CVD high risk to MI	0.0125	0.0114	0.0137	(10)
T2DM with CVD high risk to ischemic stroke	0.0065	0.0057	0.0073	(10)
T2DM with CVD high risk to unstable angina	0.0076	0.0067	0.0085	(10)
T2DM with CVD high risk to HHF	0.0080	0.0072	0.0090	(10)
T2DM with CVD high risk to CVD death	0.0070	0.0062	0.0079	(10)
T2DM with CVD high risk to Non-CVD death	0.0067	0.0059	0.0076	(10)
T2DM with MI to CVD death	0.0129	0.0106	0.0157	(20)
T2DM with MI to Non-CVD death	0.0122	0.0100	0.0150	(20)
T2DM with ischemic stroke to CVD death	0.0113	0.0097	0.0132	(10)
T2DM with ischemic stroke to Non-CVD death	0.0083	0.0069	0.0099	(10)
T2DM with unstable angina to CVD death	0.0113	0.0097	0.0132	(10)
T2DM with unstable angina to Non-CVD death	0.0083	0.0069	0.0099	(10)
T2DM with HHF to CVD death	0.0208	0.0168	0.0256	(21)
T2DM with HHF to Non-CVD death	0.0155	0.0122	0.0198	(21)

*CVD, cardiovascular disease; MI, myocardial infarction; HHF, hospitalization for heart failure; CVD death, death from cardiovascular cause; Non-CVD death, death from non-cardiovascular cause; Variation of ± 95% confidence interval was used for the transition probabilities*.

#### Cost Inputs

The study was conducted from China's health system perspective, and the cost category included direct medical costs. Direct medical costs included drug costs and treatment costs of cardiovascular events (myocardial infarction, ischemic stroke, unstable angina and hospitalization for heart failure). Because both groups of patients received standard treatment, the drug costs incurred by standard treatment were not included in the model. The cost of dapagliflozin was calculated according to the 10 mg daily doses. We assumed that the initial monitoring parameters of all patients are the same, such as HbA1c, blood lipid, blood pressure and renal function, so this part of the monitoring costs were not included in the model. In addition, the long-term management costs of cardiovascular diseases such as antihypertensive drugs, lipid-lowering drugs and antithrombotic drugs are not included in the model, so as to avoid the risk of double-counting costs. The cost of dapagliflozin was obtained from the Yaozh website (Available from: https://db.yaozh.com/, 2022). Costs associated with cardiovascular events were extracted from published China-based literature (23). Except for the cost of dapagliflozin, all costs were inflated to the 2021 EUR and CNY through the healthcare consumer price index. According to the State Administration of Foreign Exchange, in 2021, 1 EUR = 7.6369 CNY. All costs data were shown as 2021 EUR and CNY in Table 2.

**Table 2:** Cost inputs.

**Items**	**Cost (€/¥)**	**Range (€/¥)**	**References**
Annual drug costs			
Dapagliflozin	208.33/1,591	fix	Database
Standard treatment	0	–	–
Annual complication costs			
Myocardial infarction	5,459/41,690	4,094.27 ~ 6,823.78 / 31,267.50 ~ 52,112.50	(23)
Ischemic stroke	3,179/24,275	2,383.98 ~ 3,973.31 / 18,206.25 ~ 30,343.75	(23)
Unstable angina	4,145/31,657	3,108.95 ~ 5,181.59 / 23,742.75 ~ 39,571.25	(23)
Hospitalization for heart failure	3,124/23,855	2,342.74 ~ 3,904.56 / 17,891.25 ~ 29,818.75	(23)
CVD death	4,455/34,019	3,340.92 ~ 5,568.20 / 25,514.25 ~ 42,523.75	(23)
Non-CVD death	1,814/13,853	1,360.47 ~ 2,267.45 / 10,389.75 ~ 17,316.25	(23)

*Variation of ± 95% confidence interval or ± 25% of the basic data was used for the cost inputs*.

#### Utility Inputs

The health outcome of the model was quality-adjusted life-year (QALY). And the utilities were obtained from a Chinese study on deriving the health utility scores of insulin-using T2DM patients using the 3-level EQ-5D (EQ-5D-3L) questionnaire, which shown the health utility scores of different cardiovascular complications (including myocardial infarction, angina, stroke and heart failure) in Chinese patients (24). Due to that China-specific utilities study did not show the health utility score of T2DM patients with high risk of cardiovascular disease, we assumed that the health utility score of T2DM patients with high cardiovascular risk is equal to the average health utility score of all T2DM patients in the study (including patients with and without cardiovascular disease). All utility inputs were shown in Table 3.

**Table 3:** Utility inputs.

**Event**	**Value**	**Variation range**		**References**
		**Lower**	**Upper**	
T2DM with CVD high risk	0.936	0.934	0.938	(24)
T2DM complication				
Myocardial infarction	0.886	0.883	0.889	(24)
Ischemic stroke	0.830	0.827	0.833	(24)
Unstable angina	0.868	0.865	0.871	(24)
Hospitalization for heart failure	0.750	0.746	0.754	(24)

*Variation of ± 95% confidence interval was used for the utility inputs*.

### Sensitivity Analyses

One-way and probabilistic sensitivity analyses were performed to investigate the uncertainty in the model. In the one-way sensitivity analyses (DSA), relevant parameters were adjusted to their lower and upper values to identify key model input parameters that had a substantial impact on the base-case result. In this study, uncertainty ranges of ±95% confidence interval (CI) were used as the upper and lower limits of the parameters. When the value range of relevant parameters could not be obtained from the literatures, ± 25% of the basic data were used as the upper and lower limits of the parameters. The results of the one-way sensitivity analyses were presented in a Tornado diagram. Probabilistic sensitivity analysis (PSA) was used to simulate the impact of simultaneous changes in parameters in the corresponding range on the results of basic research. For the PSA, Monte Carlo simulation was used to run 1,000 iterations. Included model inputs in the PSA were costs, health utilities and transition probabilities. The gamma distribution was used for the cost parameters because of positive values, and the beta distribution was selected for the transition probability and health utility values due to the range of zero to one. The results of PSA were shown in cost-effectiveness acceptability curve and incremental cost-effectiveness scatter plot.

## Results

### Base-Case Result

The results of base-case analysis shown that in patients with T2DM and high cardiovascular risk, used of dapagliflozin plus standard treatment was predicted to result in paying more cost and getting more QALYs, compared with standard treatment alone. And the LYs of dapagliflozin plus standard treatment were predicted longer than standard treatment (13.43 LYs vs. 13.20 LYs). For patient treated with dapagliflozin plus standard treatment, the direct medical costs were €20,096.15 (¥153,472.27) and gained 12.26 QALYs. For patient treated with standard treatment, the direct medical costs were €15,660.34 (¥119,596.44) and gained 12.01 QALYs. This resulted in a predicted additional 0.25 QALYs with dapagliflozin at an incremental cost of €4,435.81 (¥33,875.83) per patient, which led to an ICER of €17,742.07 (¥135,494.41) per QALY gained. According to the WTP threshold of €31,809.77 (¥242,928), adding dapagliflozin to standard treatment is cost-effective. The predicted costs and QALYs for the two treatment regimens were shown in Table 4. The detail of stage costs, stage QALYs, cumulative costs and cumulative QALYs for the two groups were presented in Tables 5, 6.

**Table 4:** Summary of cost and outcome results from Base-Case Analysis.

**Strategy**	**Cost(€/¥)**	**Incr cost(€/¥)**	**QALY**	**Incr QALY**	**ICER (€/¥per QALY)**
Standard treatment	15,660.34/ 119,596.44	–	12.01	–	–
Dapagliflozin + standard treatment	20,096.15/ 153,472.27	4,435.81/ 33,875.83	12.26	0.25	17,742.07/ 135,494.41

*Incr Cost, increase cost; QALY, quality-adjusted life-years; Incr QALY, increase QALY; ICER, incremental cost per QALY*.

**Table 5:** Stage costs, stage QALYs, cumulative costs and cumulative QALYs for Dapagliflozin plus standard treatment group.

**Stage**	**Stage costs (¥)**	**Stage costs (€)**	**Stage QALYs**	**Cumulative costs (¥)**	**Cumulative costs (€)**	**Cumulative QALY**
0	1,112.16	145.63	0.47	1,112.16	145.63	0.47
1	2,832.07	370.84	0.88	3,944.24	516.47	1.35
2	3,605.83	472.16	0.82	7,550.07	988.63	2.17
3	4,247.09	556.13	0.77	11,797.16	1,544.76	2.94
4	4,771.87	624.84	0.72	16,569.03	2,169.60	3.66
5	5,194.49	680.18	0.68	21,763.51	2,849.78	4.34
6	5,527.72	723.82	0.63	27,291.23	3,573.60	4.97
7	5,782.94	757.24	0.59	33,074.17	4,330.84	5.56
8	5,970.30	781.77	0.56	39,044.47	5,112.61	6.12
9	6,098.81	798.60	0.52	45,143.28	5,911.20	6.64
10	6,176.47	808.77	0.49	51,319.75	6,719.97	7.12
11	6,210.41	813.21	0.46	57,530.16	7,533.18	7.58
12	6,206.91	812.75	0.43	63,737.07	8,345.93	8.01
13	6,171.57	808.12	0.40	69,908.63	9,154.06	8.40
14	6,109.30	799.97	0.37	76,017.93	9,954.03	8.78
15	6,024.45	788.86	0.35	82,042.38	10,742.89	9.13
16	5,920.86	775.30	0.33	87,963.24	11,518.19	9.45
17	5,801.89	759.72	0.31	93,765.12	12,277.90	9.76
18	5,670.48	742.51	0.29	99,435.61	13,020.41	10.05
19	5,529.23	724.02	0.27	104,964.84	13,744.43	10.31
20	5,380.39	704.53	0.25	110,345.23	14,448.96	10.57
21	5,225.91	684.30	0.23	115,571.14	15,133.25	10.80
22	5,067.50	663.55	0.22	120,638.64	15,796.81	11.02
23	4,906.62	642.49	0.20	125,545.27	16,439.30	11.22
24	4,744.54	621.27	0.19	130,289.81	17,060.56	11.41
25	4,582.33	600.03	0.18	134,872.14	17,660.59	11.59
26	4,420.92	578.89	0.17	139,293.06	18,239.48	11.76
27	4,261.09	557.96	0.16	143554.15	18,797.44	11.92
28	4,103.48	537.32	0.15	147,657.63	19,334.76	12.06
29	3,948.63	517.05	0.14	151,606.26	19,851.81	12.20
30	1,866.01	244.34	0.06	153,472.27	20,096.15	12.26

**Table 6:** Stage costs, stage QALYs, cumulative costs and cumulative QALYs for standard treatment group.

**Stage**	**Stage costs (¥)**	**Stage costs (€)**	**Stage QALYs**	**Cumulative costs (¥)**	**Cumulative costs (€)**	**Cumulative QALYs**
0	330.95	43.34	0.47	330.95	43.34	0.47
1	1,378.41	180.49	0.88	1,709.35	223.83	1.34
2	2,250.68	294.71	0.82	3,960.03	518.54	2.16
3	2,969.71	388.86	0.77	6,929.74	907.40	2.93
4	3555.00	465.50	0.72	10,484.74	1,372.91	3.65
5	4,023.83	526.89	0.67	14,508.57	1,899.80	4.32
6	4,391.54	575.04	0.63	18,900.11	2,474.84	4.95
7	4,671.71	611.73	0.59	23,571.83	3,086.57	5.53
8	4,876.35	638.52	0.55	28,448.17	3,725.09	6.08
9	5,016.05	656.82	0.51	33,464.22	4,381.91	6.59
10	5,100.18	667.83	0.48	38,564.41	5,049.75	7.07
11	5,137.00	672.65	0.45	43,701.40	5,722.40	7.52
12	5,133.75	672.23	0.42	48,835.15	6,394.63	7.93
13	5,096.80	667.39	0.39	53,931.95	7,062.02	8.32
14	5031.75	658.87	0.36	58,963.70	7,720.89	8.68
15	4,943.48	647.32	0.34	63,907.18	8,368.21	9.02
16	4,836.27	633.28	0.32	68,743.45	9,001.49	9.34
17	4,713.82	617.24	0.30	73,457.27	9,618.73	9.64
18	4,579.38	599.64	0.28	78,036.65	10,218.37	9.91
19	4,435.74	580.83	0.26	82,472.39	10,799.20	10.17
20	4,285.31	561.13	0.24	86,757.70	11,360.33	10.41
21	4,130.18	540.82	0.22	90,887.88	11,901.15	10.63
22	3,972.13	520.12	0.21	948,60.01	12,421.27	10.84
23	3,812.66	499.24	0.19	98,672.67	12,920.51	11.04
24	3,653.08	478.35	0.18	102,325.75	13,398.86	11.22
25	3,494.46	457.58	0.17	105,820.21	13,856.44	11.39
26	3,337.70	437.05	0.16	109,157.91	14,293.48	11.54
27	3,183.56	416.87	0.15	112,341.47	14,710.35	11.69
28	3,032.65	397.11	0.14	115,374.13	15,107.46	11.83
29	2,885.47	377.83	0.13	118,259.60	15,485.29	11.96
30	1,336.84	175.05	0.06	119,596.44	15,660.34	12.01

### Sensitivity Analyses

**Figure 3:**
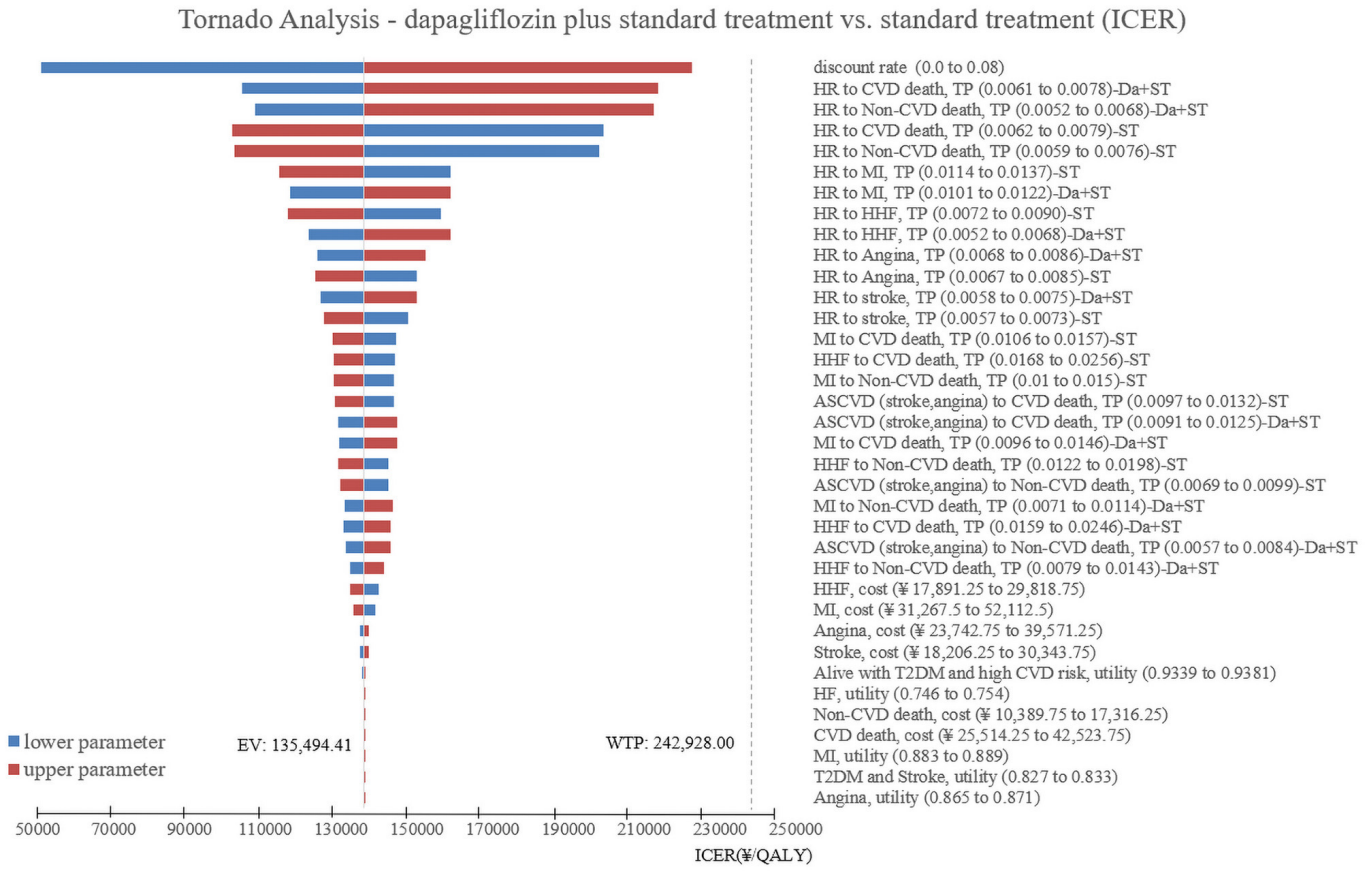
Unidirectional sensitivity analysis tornado diagram comparing the dapagliflozin plus standard treatment with standard treatment in patients with T2DM and high risk of cardiovascular disease. HR, Alive with T2DM and high CVD risk; CVD, cardiovascular disease; TP, transition probabilities; Da, dapagliflozin; ST, standard treatment; MI, myocardial infarction; HHF, hospitalization for heart failure; ASCVD, arteriosclerotic cardiovascular disease.

One-way sensitivity analysis was used to investigate the robustness of the base-case results when comparing standard treatment with dapagliflozin plus standard treatment, and then the results were summarized into tornado diagram using TreeAge 2019 software (Figure 3). The tornado diagram shown that the model results were robust when relevant parameters were adjusted one by one to their respective lower and upper values. None of the results exceeded the WTP threshold. The main driving factors of ICER included discount rate, transition probabilities for CVD death in patients with T2DM and high cardiovascular risk of dapagliflozin plus standard treatment group, and transition probabilities for Non-CVD death in patients with T2DM and high cardiovascular risk of dapagliflozin plus standard treatment group. It should be noted that when the discount rate adjusted to 0, the ICER would be changed to €6,594.77 (¥50,363.57)/QALY, which less than one time of Chinese per capita GDP (€10,603.26/¥80,976).

**Figure 4:**
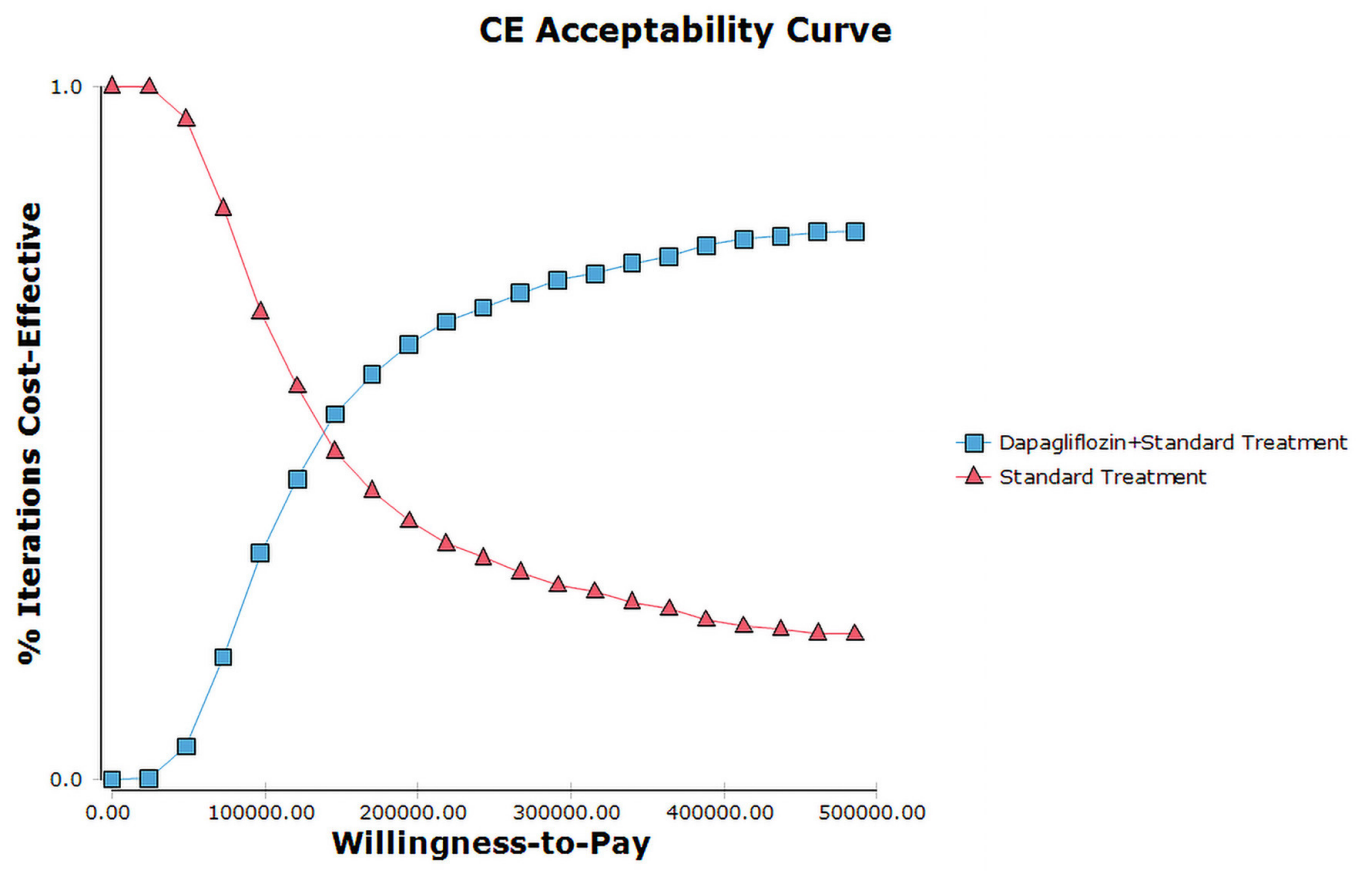
Cost-effectiveness acceptability curves for dapagliflozin plus standard treatment vs. standard treatment.

**Figure 5:**
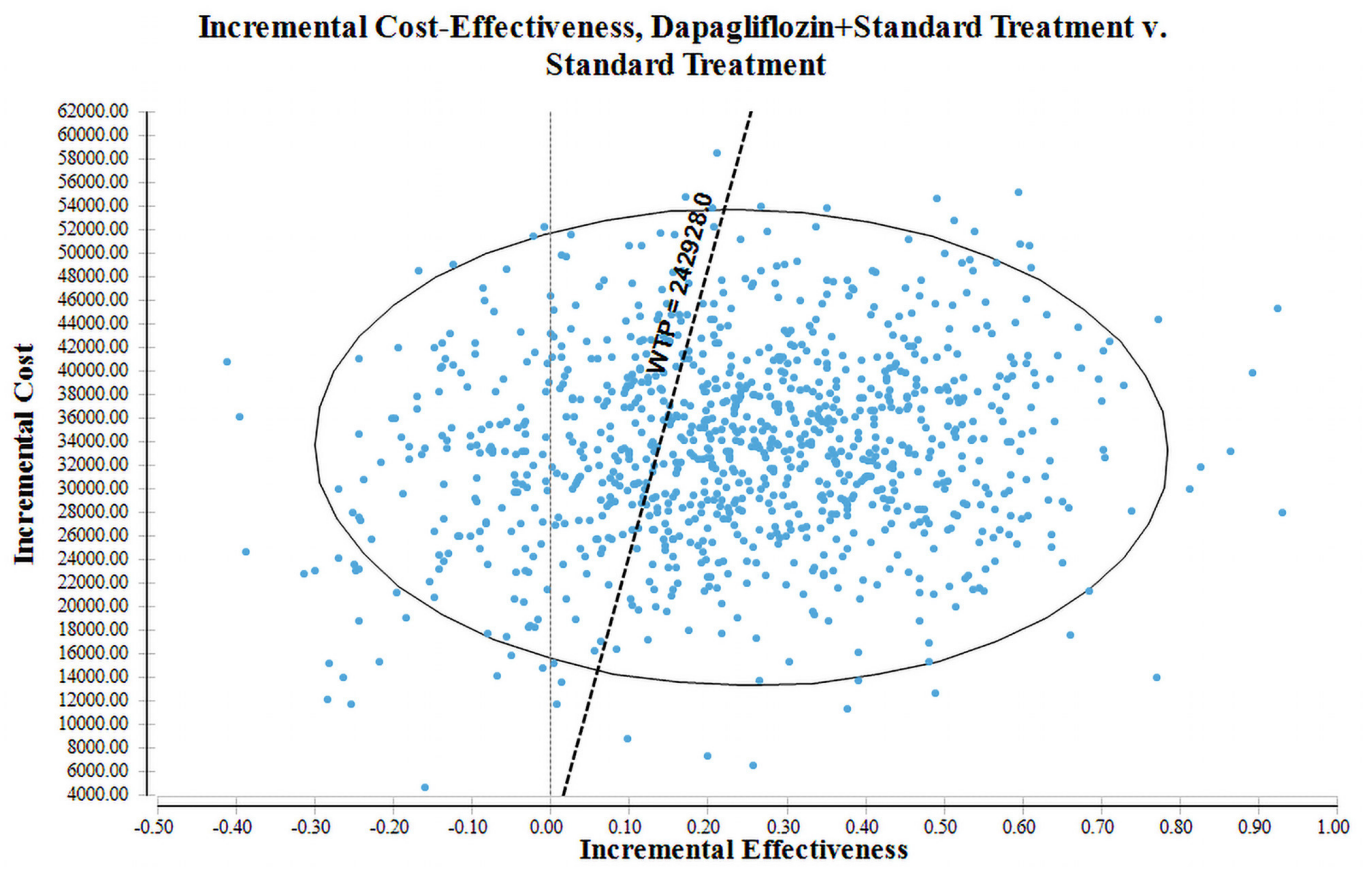
Cost-effectiveness scatter plot for dapagliflozin plus standard treatment vs. standard treatment.

The PSA with 1,000 simulations was performed by setting different distributions for each parameter to reflect the influence of the changes of all model input parameters on the base-case analysis. The results of PSA were shown in cost-effectiveness acceptability curve (Figure 4) and incremental cost-effectiveness scatter plot (Figure 5). Cost-effectiveness acceptability curve shown that when the WTP threshold was €19,085.86 (¥145,756.8), the possibility of dapagliflozin plus standard treatment being a cost-effective strategy was about 52.6%. And with the WTP threshold increased, dapagliflozin plus standard treatment was more acceptable compared to standard treatment. Incremental cost-effectiveness scatter plot shown that at three times of GDP per capita in 2021 (€31,809.77/¥242,928), dapagliflozin plus standard treatment had a 68% probability of being a cost-effective strategy.

## Discussion

Diabetes exacerbates the risk of atherosclerotic cardiovascular disease (ASCVD, including myocardial infarction, angina pectoris, stroke) and heart failure, and these cardiovascular disease increases the risk of death in diabetics (25). Therefore, it is important to select a treatment strategy that can improve the blood glucose level and the prognosis of cardiovascular disease in patients with T2DM in order to curb the progress of T2DM and its cardiovascular complications. DECLARE-TIMI 58 as the largest clinical trial to evaluate the cardiovascular outcome of dapagliflozin, proved that dapagliflozin can significantly reduce the incidence of cardiovascular death and hospitalization for heart failure in patients with T2DM and high risk of cardiovascular disease. Other results from real-world studies had also shown that patients using dapagliflozin have an overall lower risk of all-cause mortality and cardiovascular events compared with other hypoglycemic drugs (26–28). These evidences shown that dapagliflozin can be used as an important choice in the treatment of T2DM patients with high risk of cardiovascular disease.

For Chinese T2DM patients, dapagliflozin treatment was more cost-effective than metformin treatment, which was shown in the study provided by Cai et al. (29). Other researches have also shown that compared with other hypoglycemic drugs such as acarbose, glimepiride and saxagliptin, the use of dapagliflozin in Chinese T2DM patients was cost-effective (30–32). There were several studies used the data from DECLARE-TIMI 58 trial to compared the pharmacoeconomic of dapagliflozin plus standard treatment with standard treatment in patients with T2DM and high risk of cardiovascular disease. A Thai-based societal perspective analysis of dapagliflozin plus standard treatment concluded that compared with standard treatment, dapagliflozin plus standard treatment was not cost-utility. It associated with an ICER of 16,055 EUR/QALY, which exceeds the local threshold of 4,486 EUR/QALY (12). A study based on the UK setting found that dapagliflozin plus standard treatment was cost-effective. Compared with standard treatment, the patients treated with dapagliflozin plus standard treatment was estimated with an increase in QALYs from 10.43 to 10.48, and the costs were predicted to reduce from £39,451 to £36,899 (14). In Spain, compared to standard treatment, dapagliflozin as an add-on to standard treatment was cost-effect with an increase of 0.08 QALYs and at a cost saving of €2,921 (18). The conclusion could be opposite due to the differences of model structure, health systems and costs in different countries. Take the studies in setting of Thailand and UK as example, the study in Thailand perspective used Markov model to estimate the cost-effectiveness of dapagliflozin plus standard treatment. But in the study of UK setting, Cardiff T2DM cost-effectiveness model was used. The cost of dapagliflozin one patient per year use in Thailand was €410, and the local WTP was €4,486/QALY. But in UK, the cost of dapagliflozin one patient per year use was €477, the WTP in UK was € 23,256 (£20,000)/QALY. None of the results could be transferred to Chinese population. However, there was lack of pharmacoeconomic analysis of dapagliflozin plus standard treatment in Chinese patients with T2DM and high risk of cardiovascular disease. In this study, Markov model was used to evaluate the cost-effectiveness of adding dapagliflozin to standard treatment for 30 years in Chinese T2DM patients with high risk of cardiovascular disease. We used the health utility values derived from the study based on Chinese population, which included 12,583 Chinese patients from 78 hospitals nationwide. The sample was enough to cover a wide spectrum of diabetes complications. With the DECLARE-TIMI 58 results used in the model, dapagliflozin plus standard treatment was found to be cost-effective compared to standard treatment with an ICER of €17,742.07 (¥135,494.41) per QALY gained. Although the costs of adding dapagliflozin to standard treatment were higher than standard treatment, mainly due to the additional drug costs incurred by the used of dapagliflozin, these costs were also offset by the clinical benefits to patients from the used of dapagliflozin. Therefore, dapagliflozin plus standard treatment was considered to be a cost-effective therapy in patients with T2DM and high risk of cardiovascular disease. The cost-effective estimate was stable in sensitivity analyses. The evidence of this study highlights the value of dapagliflozin in clinical use and pharmacoeconomic, supports dapagliflozin as one of the first-line options for T2DM patients with high risk of cardiovascular disease in China, and provides a reference for the dynamic adjustment of the catalog of medicines covered by national medical insurance system.

The mechanism of type 2 diabetes complicated with cardiovascular disease is complex. Hyperglycemia, insulin resistance, dyslipidemia, inflammation, ROS excess, endothelial dysfunction, hypercoagulability and vascular calcification were considered participate in increasing ASCVD risk and mortality in type 2 diabetes (25). Oxidative stress, inflammation, endoplasmic reticulum (ER) stress, aberrant insulin signaling, accumulation of advanced glycated end-products, altered autophagy, changes in myocardial substrate metabolism and mitochondrial bioenergetics, lipotoxicity and altered signal transduction was related to type 2 diabetes develop heart failure (33). However, there is a literature point out that the changes of HbA1c, blood pressure and cholesterol do not seem to determine the overall benefit of SGLT2 inhibitors on cardiovascular outcomes (9). Therefore, this study chose to develop a Markov model, which depended on the endpoints data of DECLARE-TIMI 58 clinical trial to calculate the transition probabilities, instead of using an indirect model based on the risk equation of surrogate biomarkers (such as the IQVIA CORE model, which uses HbA1c, blood pressure, blood lipid and BMI index as key model parameter inputs rather than uses direct cardiovascular results from clinical trial).

However, there are still several limitations in this study. First, the medication adherence of dapagliflozin plus standard treatment therapy in the real-world may differ from in the DECLARE-TIMI 58 clinical trial. Hence, the estimates of costs and effectiveness profiles might be changed. Secondly, the health utility value used in this study was for T2DM patients using insulin in China. In the DECLARE-TIMI 58 clinical trial, the proportion of patients using insulin is about 40%. Currently, there was lack of credible study reported the utility of cardiovascular complications (such as myocardial infarction, angina and heart failure) in T2DM patients who did not use insulin in China. Sensitivity analyses attempted to overcome the uncertain to assure the base-case results. And in this study, sensitivity analyses shown that the changes of health utility values did not transform the base-case result. Thirdly, since the lack of mortality rate for patients who progression to ischemic stroke or unstable angina in the two groups, we used the results of ASCVD subgroup analysis of the DECLARE-TIMI 58 clinical trial to replace the mortality of ischemic stroke and unstable angina, which may bias the results. Fourthly, other complications, such as nephropathy and peripheral arterial disease, were not included in the model. Including them in the model will add the benefits of dapagliflozin plus standard treatment therapy (34). And in the DAPA-CKD clinical trial, compared with placebo, patients with T2DM and chronic kidney disease received dapagliflozin have a lower risk of a composite of a sustained decline in the estimated GFR of at least 50%, end-stage kidney disease, or death from renal or cardiovascular causes (35). Fifthly, the model did not include the adverse events of dapagliflozin, because they are considered transient events, and the adverse events with significant differences between the two groups were infrequent in the DECLARE-TIMI 58 clinical trial. Such as adverse event rate of genital infection in dapagliflozin group was 0.9% and in placebo group was 0.1%. Sixthly, this analysis is only applicable to T2DM patients with high risk of cardiovascular disease but not to other patients at low risk of cardiovascular disease in China. Lastly, the results of this study are specific to Chinese setting, owing to the significant differences of health systems and drug costs among countries/regions, the analysis should not be applied to other settings.

## Conclusion

In summary, from the perspective of the Chinese health system, this study suggests that dapagliflozin plus standard treatment is a cost-effective option for patients with T2DM at high cardiovascular risk. These findings may help clinicians make the best treatment decisions for patients with T2DM at high cardiovascular risk.

## Data Availability Statement

The original contributions presented in the study are included in the article/supplementary materials, further inquiries can be directed to the corresponding author/s.

## Author Contributions

KH, YW, and XX were involved in the conception and design of the study. KH, QZ, and WZ collected the data. JL and DZ provided suggestions for model structure. KH and SS made the data visualization. KH and YW developed the model. KH wrote the first draft of the manuscript and performed the health economic analysis. XX revised the manuscript critically for important intellectual content. All authors contributed to the article and approved the submitted version.

## Funding

This work was supported by The University Synergy Innovation Program of Anhui Province (grant number: GXXT-2021-068).

## Conflict of Interest

The authors declare that the research was conducted in the absence of any commercial or financial relationships that could be construed as a potential conflict of interest.

## Publisher's Note

All claims expressed in this article are solely those of the authors and do not necessarily represent those of their affiliated organizations, or those of the publisher, the editors and the reviewers. Any product that may be evaluated in this article, or claim that may be made by its manufacturer, is not guaranteed or endorsed by the publisher.
